# Hexestrol Deteriorates Oocyte Quality via Perturbation of Mitochondrial Dynamics and Function

**DOI:** 10.3389/fcell.2021.708980

**Published:** 2021-07-06

**Authors:** Dong Niu, Kun-Lin Chen, Yi Wang, Xiao-Qing Li, Lu Liu, Xiang Ma, Xing Duan

**Affiliations:** Key Laboratory of Applied Technology on Green-Eco-Healthy Animal Husbandry of Zhejiang Province, College of Animal Science and Technology, College of Veterinary Medicine, Zhejiang A&F University, Hangzhou, China

**Keywords:** hexestrol, ovary, oocyte maturation, spindle, mitochondria, apoptosis

## Abstract

Hexestrol (HES) is a synthetic non-steroidal estrogen that was widely used illegally to boost the growth rate in livestock production and aquaculture. HES can also be transferred to humans from treated animals and the environment. HES has been shown to have an adverse effect on ovarian function and oogenesis, but the potential mechanism has not been clearly defined. To understand the potential mechanisms regarding how HES affect female ovarian function, we assessed oocyte quality by examining the critical events during oocyte maturation. We found that HES has an adverse effect on oocyte quality, indicated by the decreased capacity of oocyte maturation and early embryo development competency. Specifically, HES-exposed oocytes exhibited aberrant microtubule nucleation and spindle assembly, resulting in meiotic arrest. In addition, HES exposure disrupted mitochondrial distribution and the balance of mitochondrial fission and fusion, leading to aberrant mitochondrial membrane potential and accumulation of reactive oxygen species. Lastly, we found that HES exposure can increase cytosolic Ca^2+^ levels and induce DNA damage and early apoptosis. In summary, these results demonstrate that mitochondrial dysfunction and perturbation of normal mitochondrial fission and fusion dynamics could be major causes of reduced oocyte quality after HES exposure.

## Introduction

Environmental pollution is becoming a major threat to human health. One emerging group of contaminants, endocrine-disrupting chemicals (EDCs), is exogenous substances or mixtures that cause dysfunction of the endocrine system, inducing multiple adverse health effects in humans through interaction with hormone receptors (Schug et al., 2016). EDCs can be released into the environment in various forms, including pesticides, plastic debris, retardants, industrial waste, and cosmetics (Chen et al., 2019; Canipari et al., 2020), and they can be absorbed, accumulated, and even converted to more toxic metabolites by the body (Yuan et al., 2015; Ding et al., 2020). Studies have demonstrated that EDCs affect the reproductive systems of both sexes, causing congenital abnormalities and infertility (Huang and Zeng, 2021; Walker et al., 2021; You and Song, 2021). In female mammals, EDCs usually affect hormone–receptor interactions, corpus luteum formation, sex steroid synthesis, and folliculogenesis, resulting in irreversible reproductive issues such as estrogen deficiency, dysfunctional ovulation, premature ovarian insufficiency, endometriosis, polycystic ovarian syndrome, or infertility (Craig et al., 2011; Hamid et al., 2021).

Hexestrol (HES) is a synthetic hormone belonging to a class of non-steroidal estrogens and is used in clinics for the treatment of prostate cancer, amenorrhea, uterine hypoplasia, dysfunctional uterine bleeding, and menopausal syndrome (Jan et al., 1998; Lucan et al., 2013). However, because of its adverse effects on human health and the reproductive system, HES for human use has been banned. Nevertheless, HES is still illegally used as a growth hormone in the agricultural industry to increase the weight of animals by promoting protein synthesis and inhibiting fat aggregation (Xiangqian et al., 2007; Gao et al., 2015; Feng et al., 2016), which may contribute to HES exposure in the environment. Thus, humans are not only exposed to environmental HES directly, but it can also pass through the food chain to the human body, and it interferes with normal physiological processes, resulting in severe health problems such as cancer, endometriosis, fetal malformation, and metabolic disorders (Saeed et al., 2005; Cavalieri and Rogan, 2006; Chen et al., 2015; Feng et al., 2016).

The prevailing adverse effect of HES exposure is its severe adverse effects on reproductive function. HES exposure for 4 weeks can induce degeneration of spermatocytes and cause infertility in male rabbits (Pop et al., 2011). In addition to its adverse effects on male reproduction, HES also has adverse effects on the female reproductive system. HES exposure can increase the expression of growth hormone-releasing hormone by activating P38 and cyclic AMP response element-binding protein in placental cells, which may cause the dysregulation of placental hormones and abortion (Zhu et al., 2016). The reproductive toxicity of HES has also been observed in adult female mice, which showed decreased ovary size and impaired development of primordial follicles (de Oliveira et al., 2008). A previous study demonstrated that clomiphene citrate, a non-steroidal triphenylethylene compound, has an adverse effect on gonadotropin-induced ovulation by reducing cyclic AMP and prostaglandin E2 levels in the ovary (Chaube et al., 2005, 2006). However, the mechanism by which HES affects ovary function and oocyte quality has not yet been clarified.

Oocyte maturation is involved in various developmental events and regulators that drive oocyte nuclear maturation and cytoplasmic maturation, which is critical for fertilization and embryonic development. During meiosis, precise regulation of spindle assembly and accurate chromosome segregation ensure the integrity of the genome throughout embryonic development (Clift and Schuh, 2013). Abnormal spindle assembly and chromosome segregation usually cause aneuploidy and spontaneous abortion in mammals (Webster and Schuh, 2017). The morphology and distribution of organelles, such as mitochondria, also play important roles during oocyte maturation, including the synthesis of adenosine triphosphate, maintenance of Ca^2+^ homeostasis, and regulation of redox. Mitochondrial dysfunction can cause excessive production of reactive oxygen species (ROS) and disrupt redox balance, inducing oxidative stress and cell apoptosis and impaired developmental competence of oocytes (Chiang et al., 2020; Richani et al., 2021).

In the present study, we used mouse oocytes as a research model to investigate the impact of HES exposure on oocyte maturation and fertilization. We observed that HES exposure caused aberrant oocyte meiotic progression by inducing abnormal spindle assembly and imbalanced mitochondrial fission and fusion. Additionally, we observed that HES exposure disrupted Ca^2+^ homeostasis and induced DNA damage and apoptosis in oocytes. These data will expand our knowledge of how HES affects female gamete development.

## Materials and Methods

### Mouse Oocyte Preparation and Culture

Mice were treated and used according to the guidelines of the Institutional Animal Care and Use Committee at Zhejiang A&F University, China. For *in vivo* experiment, 30 mice were randomly divided into two groups (body weight: 20–21 g per mice) and housed at controlled condition temperature (24°C) and light (12-h light–dark cycle). The mice were continuously treated by oral gavage administration of 0 and 6 mg/kg/day of HES (dissolved in corn oil, 100 μl/day) for 1 month. For *in vitro* experiment, HES were dissolved in DMSO to prepare a 100-mM stock solution and then diluted in an M16 medium at different concentrations for an experiment. Based on the effect of HES on the first polar body extrusion, 100 μM was chosen for the following experiments. In all experiments, GV oocytes were collected at room temperature from 6 to 8-week-old female ICR mice after injecting with pregnant mare serum gonadotropin (5 IU). Oocytes were kept in an M2 medium containing 3-isobutyl-1-methylxanthine during collection and cultured in a 3-isobutyl-1-methylxanthine-free M16 medium (Sigma-Aldrich) under mineral oil at 37°C in a 5% carbon dioxide atmosphere.

### Immunofluorescence Staining and Confocal Microscopy

Oocytes were fixed in 4% paraformaldehyde in phosphate-buffered saline (PBS) for 30 min and then transferred into a membrane permeabilization solution (0.5% Triton-X-100 in PBS) for 20 min at room temperature. After blocking with 1% bovine serum albumin in PBS for 1 h, the oocytes were stained with primary antibody overnight at 4°C [dynamin-related protein 1 (Drp1) 1:100; p-Drp1 1:100; targeting-protein for Xklp2 (TPX2) 1:100; pericentrin 1:200; γH2AX 1:200; α-tubulin-fluorescein isothiocyanate (FITC) 1:200], then subjected to secondary antibody staining for 1 h. Oocytes were washed three times with 0.1% Tween 20, 0.01% Triton-X-100 in PBS, stained with Hoechst 33342 (10 μg/ml in PBS) for 10 min. Then, oocytes were subjected to confocal microscopy imaging (FV3000, Olympus). Both control and HES-treated oocytes were scanned with the same parameters under confocal microscopy; the images were analyzed by Image J software (NIH, Bethesda, MD, United States).

### Mitochondrial Distribution and Membrane Potential Assay in Oocytes

Mitochondrial distribution and mitochondrial membrane potential were examined using MitoTracker Red or MitoProbe JC-1. Briefly, the oocytes in each group were exposed to 500-nM MitoTracker Red or 50 μg/ml MitoProbe JC-1 for 30 min at 37°C and then washed with the M2 medium twice. After that, the oocytes were subjected to confocal microscopy imaging.

### Detection of Reactive Oxygen Species Level in Oocytes

To determine intracellular ROS levels, oocytes were incubated with 10-μM dichlorofluorescein diacetate for 30 min at 37°C and washed three times with the M2 medium. Then, the fluorescent signal of oocytes was scanned with the same parameters under confocal microscopy (Jia et al., 2019).

### Intracellular Calcium and Apoptosis Assay in Oocytes

Ca^2+^ probe Fluo 4-AM measured intracellular calcium in oocytes. Briefly, the oocytes were incubated with 2 μM Fluo 4-AM for 30 min at 37°C (Liu et al., 2019); after washing three times with PBS, the oocytes were subjected to confocal microscopy imaging. For the apoptosis assay, live oocytes were incubated with a 45 μl binding buffer containing 5 μl of annexin-V-FITC for 30 min at 37°C; after washing three times with PBS (Zhou et al., 2019), oocytes were subjected to confocal microscopy imaging.

### Antibodies and Chemicals

Rabbit monoclonal anti-DRP1 (D6C7), rabbit polyclonal anti-phospho-DRP1 (Ser616), and rabbit monoclonal anti-phospho-histone H2A.X antibodies were purchased from Cell Signaling Technology (Danvers, MA, United States); mouse monoclonal anti-α-tubulin-FITC and HES were obtained from Sigma-Aldrich (St. Louis, MO, United States); rabbit polyclonal TPX2 antibody was obtained from Novus Biologicals (Colorado, United States); mouse polyclonal anti-pericentrin antibody was purchased from BD Biosciences (San Jose, CA).

### Immunoblotting

Oocytes were lysed in 1 × lithium dodecyl sulfate sample buffer (Thermo Fisher, Waltham, MA, United States) and heated at 95°C for 10 min. Protein was separated on 4–20% sodium dodecyl sulfate–polyacrylamide gel electrophoresis mini gels (Thermo Fisher, Waltham, MA, United States) and blotted to polyvinylidene fluoride membranes (Millipore). The blots were incubated for 1 h with 5% non-fat milk in Tris-buffered saline, 0.05% Tween-20, and then incubated overnight with primary antibodies diluted in tyrosylprotein sulfotransferase; after incubation with secondary antibodies for 1 h, the blots were washed with Tris-buffered saline, 0.05% Tween-20 three times. Chemiluminescence signals were developed with SuperSignal West Femto Kit (Thermo Fisher Scientific, Waltham, MA, United States) and were captured by Tanon-5200 imaging system.

### Hematoxylin and Eosin Staining

The ovaries were fixed in 4% paraformaldehyde in PBS for more than 24 h at room temperature. After dehydrating with graded ethanol and clearing in xylene, the ovaries were embedded in paraffin; then, the samples were sliced on 3-μm thickness by a microtome and stained with hematoxylin and eosin (H&E). The sections were examined with an optical microscope (Olympus, IX73).

### Quantification of Immunofluorescence Imaging

The immunofluorescence (IF) imaging was analyzed by Fiji software. The area of the spindle was measured on maximum z-projections when the spindle was segmented by an intensity threshold. For quantification of the number of pericentrin foci, images were manually removed with the out-of-cell non-specific signal by image J. Specific method is as follows: (1) median filter, radius two pixels; (2) auto-threshold by moments, dark; (3) convert to mask; (4) run watershed; (5) analyze particles; (6) display results. The number and size of foci were statistically analyzed by GraphPad 8.

### *In vitro* Fertilization and Embryonic Culture

Female ICR mice (6–8 weeks) were super-ovulated by intraperitoneal injection of pregnant mare’s serum gonadotropin (5 IU); then, the mice were injected with 5-IU human chorionic gonadotrophin after 48 h. Ovulated cumulus–oocytes complexes were collected from the ampulla of the fallopian tube and placed in human tubal fluid (HTF) after 13–15 h. Sperm was isolated from adult male epididymis and pre-incubated in HTF for 1 h to capacitate. Dispersed spermatozoa were added to HTF drops containing ovulated cumulus–oocytes complex for 6 h at 37°C in a 5% carbon dioxide atmosphere. Zygotes were washed and transferred to a potassium simplex optimized medium and cultured to the morular/blastocyst stage.

### Statistical Analysis

The statistical analysis was performed with GraphPad Prism software 8.0.1 (La Jolla, United States). Comparisons of data were performed by one-way analysis of variance with Turkey’s multiple comparisons.

## Results

### Hexestrol Affects Relative Ovary Weight and Morphology

**FIGURE 1:**
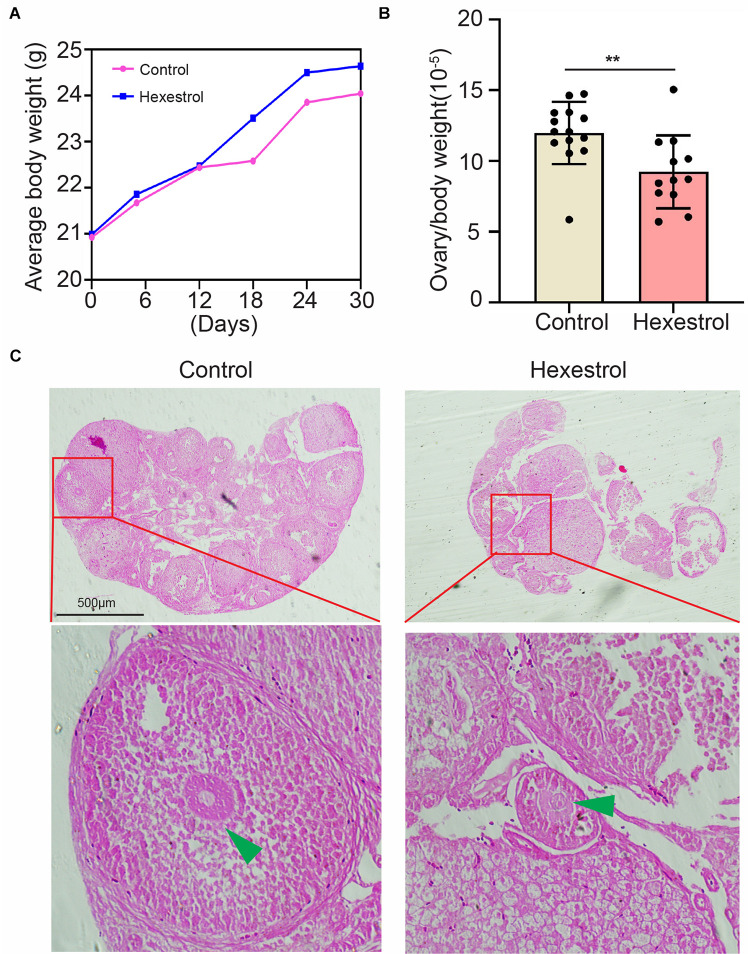
HES affects mouse ovary weight and morphology. **(A)** Compared with control group, body weight was slightly though not significantly increased. **(B)** Ovary weight was significantly reduced after exposure to HES. **P = 0.0074. Data are represented as mean ± SD from at least three independent experiments. **(C)** Representative H&E histochemical section images of ovaries from control and HES-exposed groups. Scale bar, 500 μm.

To identify the effect of HES on female reproduction, the body weight and ovary morphology of adult female mice were examined after 30 days of exposure to HES. As shown in Figure 1A, body weight was continuously monitored on days 6, 12, 18, 24, and 30. The results indicate that HES exposure for 1 month slightly increased the body weight. However, ovary weight was remarkably reduced after HES treatment compared with that of the control group (Figure 1B). To better understand the effect of HES on ovary function, we performed H&E staining to determine the morphology of the ovary structure (Figure 1C). We observed that the structure of mature follicles was impaired, demonstrating that HES exposure had an adverse effect on ovary function and folliculogenesis.

### Hexestrol Impairs Oocyte Maturation and Early Embryonic Development

**FIGURE 2:**
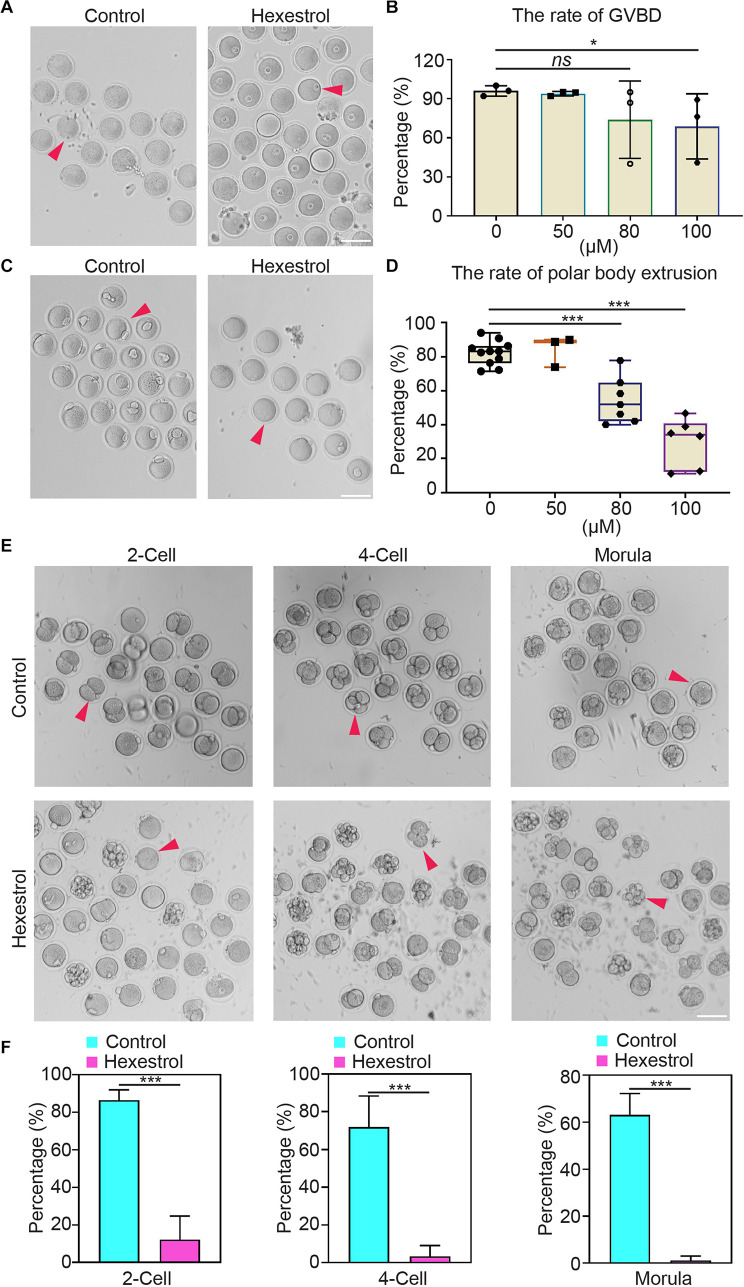
HES impairs oocyte maturation and early embryonic development. **(A)** Representative images of germinal vesicle breakdown oocytes in control and HES-exposed oocytes. Scale bar, 100 μm. **(B)** Percentage of germinal vesicle breakdown was quantified in control and in oocytes exposed to different concentrations of HES (50, 80, and 100 μM). ns, *P >* 0.05, and *P < 0.05. **(C)** Representative images of first polar body extruded-oocytes from control and HES-exposed groups. Scale bar, 100 μm. **(D)** Rate of polar body extrusion was recorded in control and HES-exposed groups. ***P < 0.001. **(E)** Representative images of fertilized eggs in control and HES-exposed group. Scale bar, 100 μm. **(F)** Early embryonic development was recorded in control and HES-exposed group. ***P < 0.001. Data are represented as mean ± SD from at least three independent experiments.

To determine the influence of HES on oocyte quality, oocyte meiotic progression was investigated by quantifying the occurrence of germinal vesicle breakdown and first polar body extrusion after exposure to different concentrations of HES *in vitro*. The quantitative results showed that HES exposure significantly reduced the percentage of germinal vesicle breakdown and the first polar body extrusion in a dose-dependent manner, particularly at 100 μM, suggesting that HES may inhibit the meiotic maturation of mouse oocytes (Figures 2A–D). Accordingly, we then used 100 μM of HES for the following experiments.

Next, we examined the effects of HES exposure on early embryonic development. Most of the zygotes developed into two-cell embryos in the control group, but the frequency was remarkably reduced after exposure to HES. Consistently, the percentages of four-cell embryos and morula were significantly decreased compared with those of the control group (Figures 2E,F). The observations mentioned earlier suggest that HES exposure has an adverse effect on oocyte maturation and early embryonic developmental competence.

### Hexestrol Disturbs Microtubule Assembly and the Coalescence of Microtubule-Organizing Centers in Metaphase I Spindle Poles

**FIGURE 3:**
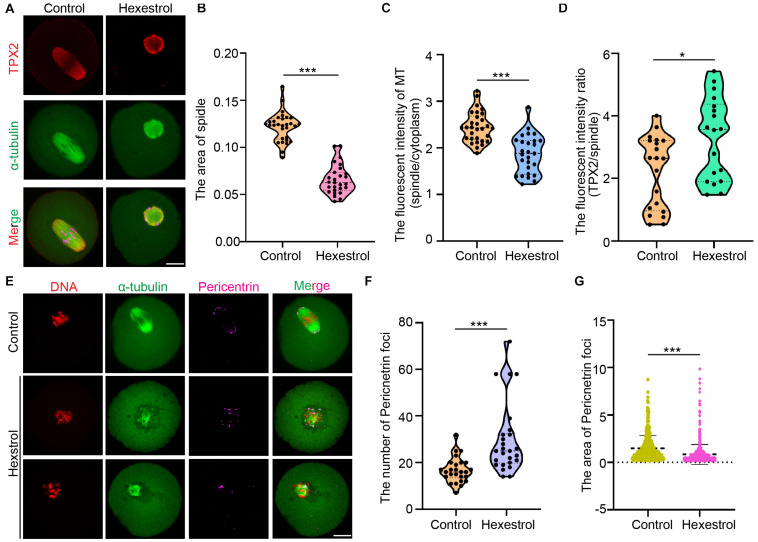
HES disturbs MT assembly and coalescence of MTOCs in MI spindle poles. *P* < 0.001. **(A)** Representative images of TPX2 localization in control and HES-exposed oocytes. Scale bar, 20 μm. **(B)** Spindle area was quantified in control and HES-exposed oocytes. ***P < 0.001. **(C)** Ratio of MT fluorescent intensity in spindle and cytoplasm was quantified in control and HES-exposed oocytes. ***P < 0.001. **(D)** Ratio of TPX2/spindle fluorescent intensity was recorded in control and HES-exposed oocytes. *P < 0.05 and ***P < 0.001. **(E)** Representative images of spindle morphologies and pericentrin localization in control and HES-exposed oocytes. Scale bar, 20 μm. **(F,G)** Number and area of pericentrin foci were quantified in control and HES-exposed oocytes. ***P < 0.001. Data are represented as mean ± SD from at least three independent experiments.

Given that cytoskeletal organization is usually associated with oocyte maturation, we considered whether HES exposure has an adverse influence on a spindle assembly. We analyzed confocal IF images of oocytes fixed and stained after HES exposure. The results showed that HES exposure significantly reduced spindle-associated tubulin and spindle size and also induced the formation of multipolar spindles, indicating an overall decrease in spindle microtubule (MT) stability. Interestingly, HES exposure resulted in the loss of more tubulin than TPX2 from the spindles, indicating that MT-bound TPX2 could stabilize the already formed spindle MTs. In addition, we noticed that HES exposure induced the formation of ectopic MT asters, indicating that HES could disrupt the coalescence of microtubule-organizing centers (MTOCs) during metaphase I (MI) spindle assembly (Figures 3A–D). To test this, we used IF staining to examine the effect of HES on pericentrin. We observed that the number of MTOCs visualized as pericentrin foci was significantly increased in oocytes. However, the average size was reduced in HES-exposed oocytes (Figures 3E–G). Therefore, our results indicate that HES exposure not only inhibits spindle MT nucleation and assembly but also regulates MI spindle pole formation by regulating the coalescence of pericentrin-containing MTOCs in MI oocytes.

### Hexestrol Affects Mitochondrial Function in Oocytes

**FIGURE 4:**
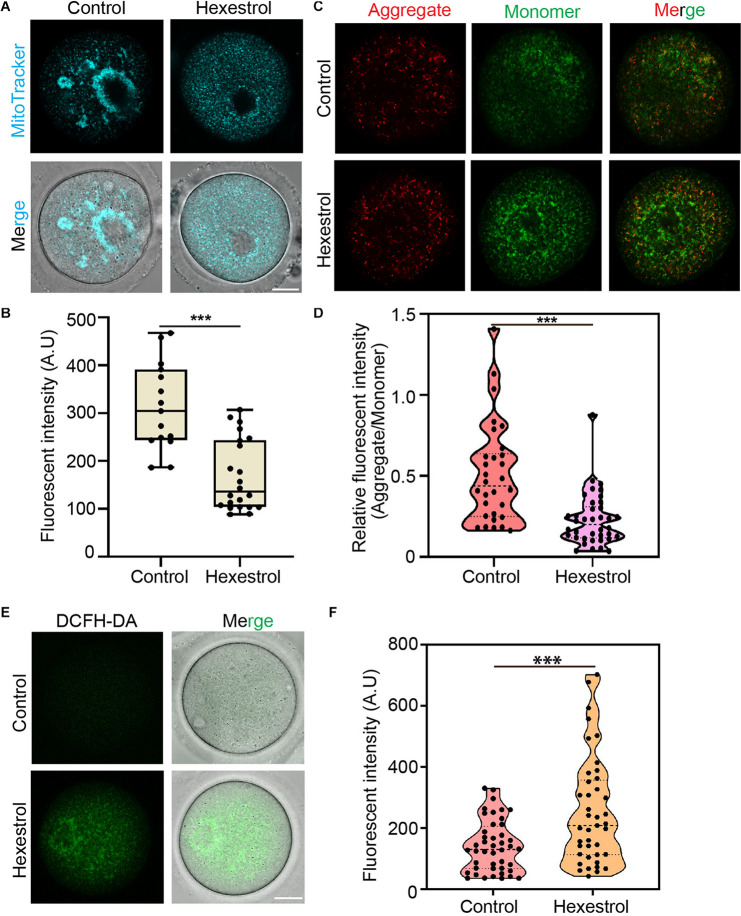
HES affects mitochondrial function in oocytes. **(A)** Mitochondria distribution was examined in control and HES-exposed oocytes. Scale bar, 20 μm. **(B)** Fluorescent intensity of Mito-Tracker was quantified in control and HES-exposed oocytes. ***P < 0.001. **(C)** Representative images of JC-1 kit staining in control and HES-exposed oocytes. Scale bar, 20 μm. **(D)** Mitochondrial membrane potential was recorded after HES exposure (Aggregate/Monomer). ***P < 0.001. **(E)** Representative images of ROS levels in control and HES-exposed oocytes. Scale bar, 20 μm. **(F)** Fluorescent intensity of ROS was analyzed in control and HES-exposed oocytes. ***P < 0.001. Data are represented as mean ± SD from at least three independent experiments.

Given the critical function of mitochondria during oocyte maturation, we assessed mitochondrial distribution using MitoTracker staining. As shown in Figure 4A, mitochondria mainly accumulated around the spindle in the control oocytes. Conversely, HES exposure induced the homogeneous distribution of mitochondria in the cytoplasm of oocytes, and the quantitative results indicated that the fluorescence intensity of MitoTracker was significantly reduced in HES-treated oocytes (Figure 4B), which demonstrated that HES has an adverse effect on mitochondrial biogenesis. To better understand its effect on mitochondrial function, we performed JC-1 staining to examine the mitochondrial membrane potential, which is an important criterion for evaluating mitochondrial activity and function. As shown in Figure 4C, HES exposure increased the formation of monomers in oocytes, indicating a low mitochondrial membrane potential. Consistently, the quantitative results demonstrated that the fluorescent intensity of the aggregate/monomer was remarkably reduced in HES-exposed oocytes compared with that of controls (Figure 4D). The dysfunction of mitochondria usually accompanied by the generation of ROS and induction of oxidative stress, which perturbs the normal cellular function. Accordingly, we detected ROS levels after HES exposure by 2′–7′dichlorofluorescin diacetate staining. As expected, ROS levels were prominently increased in HES-exposed oocytes compared with control oocytes (Figures 4E,F).

### Hexestrol Perturbs Mitochondrial Fission and Fusion Processing Oocytes

**FIGURE 5:**
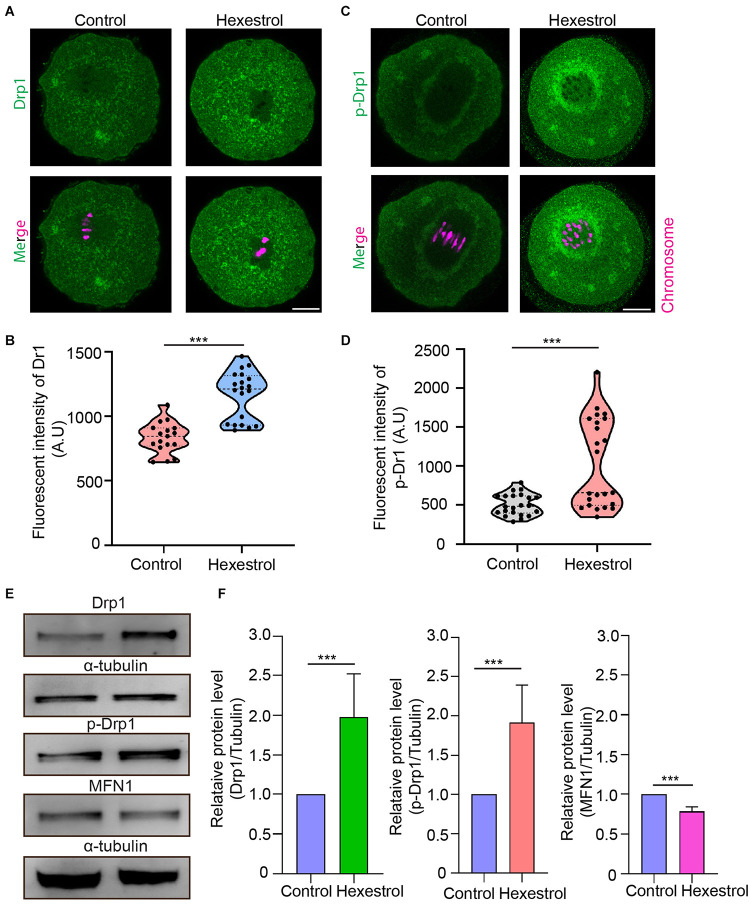
HES perturbs mitochondrial fission and fusion process in oocytes. **(A)** Representative images of Drp1 localization in control and HES-exposed oocytes. Scale bar, 20 μm. **(B)** Fluorescent intensity of Drp1 was quantified after HES exposure. ***P < 0.001. **(C)** Representative images of phosphorylated-Drp1 localization in control and HES-exposed oocytes. Scale bar, 20 μm. **(D)** Fluorescent intensity of p-Drp1 was recorded after HES exposure. ***P < 0.001. **(E)** Drp1, p-Drp1, and MFN1 protein levels were examined by using Western blot. Data are represented as mean ± SD from at least three independent experiments. **(F)** The protein levels of Drp1, p-Drp1, and MFN1 were quantified by Image J.

The abnormal mitochondrial distribution and morphology indicated that mitochondrial fission and fusion processes are likely to be affected. To test this, we assessed the expression level and phosphorylation of Drp1, a key factor in mediating mitochondrial fission, after HES exposure by immunofluorescent staining to evaluate the balance of mitochondrial fission and fusion processes. The fluorescence images and intensity measurement results indicated that HES exposure could increase mitochondrial fission by increasing Drp1 expression and phosphorylation of Drp1 during oocyte meiosis (Figures 5A–D). Consistently, the Western blot data also verified that HES exposure disrupted normal mitochondrial fission and fusion balance. Correspondingly, the protein level of mitofusin 1, a key factor of regulating mitochondrial fusion, was significantly reduced after exposure to HES in oocytes (Figures 5E,F). Taken together, our data demonstrated that the imbalance of mitochondrial fission and fusion induced by HES exposure compromised mitochondrial function, resulting in oocyte meiotic defects and the failure of early embryonic development.

### Hexestrol Increases Intracellular Calcium Level and DNA Damage to Induce Oocyte Apoptosis

**FIGURE 6:**
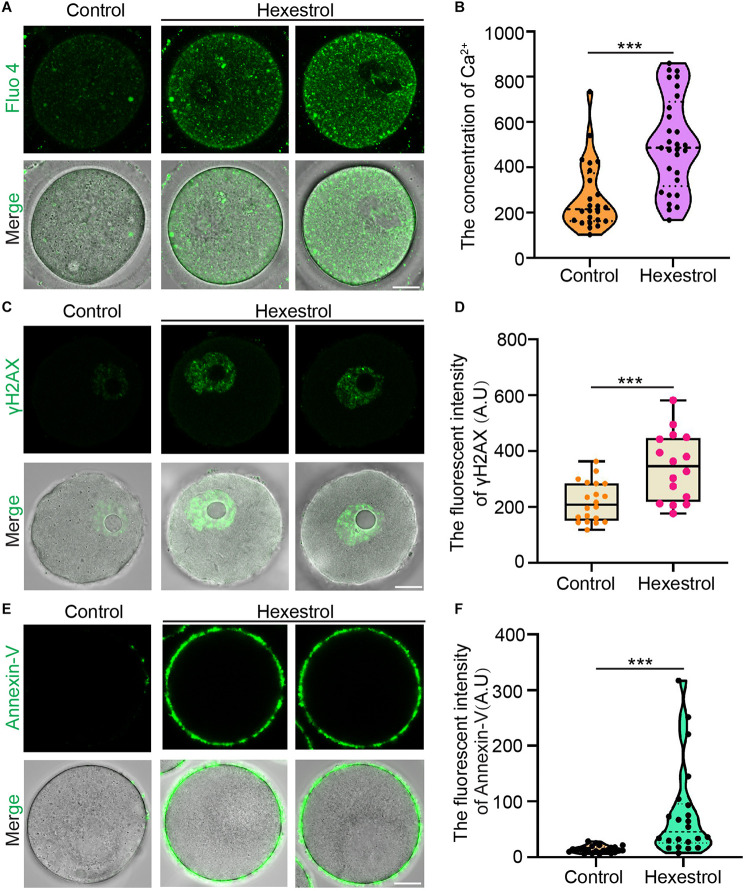
HES increases intracellular calcium level and DNA damage to induce oocyte apoptosis. **(A)** Representative images of Ca^2+^ levels in control and HES-exposed oocytes. Scale bar, 20 μm. **(B)** Fluorescent intensity of Ca^2+^ was quantified in control and HES-exposed oocytes. ***P < 0.001. **(C)** Representative images of γH2AX localization in control and HES-exposed oocytes. Scale bar, 20 μm. **(D)** Fluorescent intensity of γH2AX was recorded in control and HES-exposed oocytes. ***P < 0.001. **(E)** Representative images of apoptotic oocytes in control and HES-exposed oocytes. Scale bar, 20 μm. **(F)** Fluorescent intensity of annexin-V was quantified in control and HES-exposed oocytes. ***P < 0.001. Data are represented as mean ± SD from at least three independent experiments.

Because mitochondrial dysfunction can disturb calcium homeostasis, we next evaluated the intracellular Ca^2+^ level by staining with Fluo-4 dye. Fluorescence imaging and quantitative data indicated that the intracellular Ca^2+^ level was significantly increased after exposure to HES (Figures 6A,B). Dysfunction related to ROS produced by mitochondria usually leads to an accumulation of DNA damage and apoptosis. We, therefore, next examined the effect of HES on oocyte DNA damage by γH2AX staining and early apoptosis using annexin-V staining. As expected, HES exposure resulted in significantly increased fluorescent signals of γH2AX and annexin-V (Figures 6C–F). In summary, these results demonstrate that dysfunction of mitochondria caused by HES exposure can disrupt the homeostasis of calcium and induce the generation of excessive ROS, causing DNA damage and early apoptosis, thereby resulting in low oocyte quality.

## Discussion

Widely used EDCs in industrial products have attracted attention because of their adverse effects on human health, especially on the reproductive system (Green et al., 2021). A previous study showed that HES has toxic effects on adult female mice, resulting in a reduced number of ovarian follicles and disruptions to ovarian morphology (de Oliveira et al., 2008). However, it has not yet been established whether HES has an adverse effect on oocyte quality and female fertility. In this study, we investigated the effects of HES on mouse oocyte maturation and early embryonic development.

We first found that HES exposure resulted in a decrease in ovary weight and abnormal ovary structure, confirming the adverse effect of HES on the female reproductive system (Inazu and Satoh, 1994; de Oliveira et al., 2008). To better understand the toxicity to female reproductive function, we examined the capacity for oocyte maturation and early embryonic development. The results revealed that HES exposure led to oocyte meiotic arrest at the MI stage by disrupting the extrusion of the first polar body. Further results indicated that meiotic failure in HES-exposed oocytes was caused by aberrant morphology of spindles in the MI stage. The spindle assembly checkpoint monitors the attachment of spindle MTs and kinetochores to protect oocytes from chromosome missegregation and aneuploidy (Marston and Wassmann, 2017). Aberrant spindle MT dynamics could activate spindle assembly checkpoint and arrested oocytes at the MI stage. Previous studies have shown that MTOCs are essential for spindle formation in mammalian oocytes, including γ-tubulin, pericentrin, and its partner CDK5RAP2 (Carabatsos et al., 2000; Combelles and Albertini, 2001; Balboula et al., 2016; Baumann et al., 2017). More importantly, pericentrin plays a crucial role as a scaffolding protein that brings together many other MTOC constituents, which regulate meiotic spindle assembly. Therefore, aberrant coalescence of pericentrin can induce defects in spindle organization (Ma and Viveiros, 2014; Drutovic et al., 2020). Our current observations showed that HES exposure resulted in an increased number of pericentrin foci dispersed in MI oocytes and explained the reason for HES-induced aberrant spindle morphology and meiotic arrest. It has been reported that TPX2 is critical for chromatin-mediated MT nucleation under the control of Ran GTPase (Helmke and Heald, 2014). The reduction of spindle area in HES-exposed oocytes led us to speculate that HES treatment caused the defects in MT nucleation. Our results showed that HES exposure could increase MT-bound TPX2 to resist HES-induced disassembly of MTs in MI oocytes, contributing to the low sensitivity of MI spindle MTs to HES.

In mammals, mitochondria are dynamic organelles that play critical roles in cellular energy metabolism and redox homeostasis in the oocyte (Marei et al., 2019; Chiang et al., 2020; Soto-Heras and Paramio, 2020). During oocyte maturation, mitochondria constantly undergo fission and fusion processes, essential for maintaining mitochondrial distribution in MI oocytes (Duan et al., 2020). Recent studies have shown that an imbalance in mitochondrial fission and fusion processes is associated with the low quality of oocytes. Inhibition of mitochondrial fission by Drp1 depletion causes aberrant organelle distribution and the failure of spindle migration (Udagawa et al., 2014; Duan et al., 2020). Moreover, depletion of mitofusin 1 modulates mitochondrial function and oocyte developmental competence in oocytes (Hou et al., 2019). As expected, our findings revealed that HES exposure disrupted the mitochondrial distribution and the balance of mitochondrial fission and fusion process, which might lead to defects in the spindle during oocyte maturation, resulting in embryo death. The destruction of mitochondria network dynamics is usually associated with mitochondrial dysfunction, accompanied by the generation of excessive ROS and a reduction in mitochondrial membrane potential (Sabouny and Shutt, 2020). In concordance with this, our results demonstrated that HES exposure strongly reduced mitochondrial membrane potential and increased ROS generation in oocytes.

In addition to the defects in mitochondrial function, we also discovered that HES exposure led to an increase in cytosolic Ca^2+^ concentration in MI oocytes. It has been reported that a reduction in cytosolic Ca^2+^ levels can prevent mitochondrial fragmentation (Pinton et al., 2001; Tiwari et al., 2017a); this could explain why the defect in mitochondrial fission and fusion processes might be caused by the increase of cytosolic Ca^2+^ in oocytes; however, the detailed mechanism needs to be further explored. More evidence has demonstrated that oxidative stress can induce DNA damage and early apoptosis (Zhang et al., 2019; Kello et al., 2020; Sun et al., 2020; Shi et al., 2021), resulting in a decline in oocyte quality and fertilization. In line with previous studies (Chaube et al., 2008; Tiwari et al., 2017b), we found that oocytes exposed to HES induced the accumulation of DNA damage and the occurrence of early apoptosis, which explains HES-induced decline in mouse oocyte quality.

## Conclusion

**FIGURE 7:**
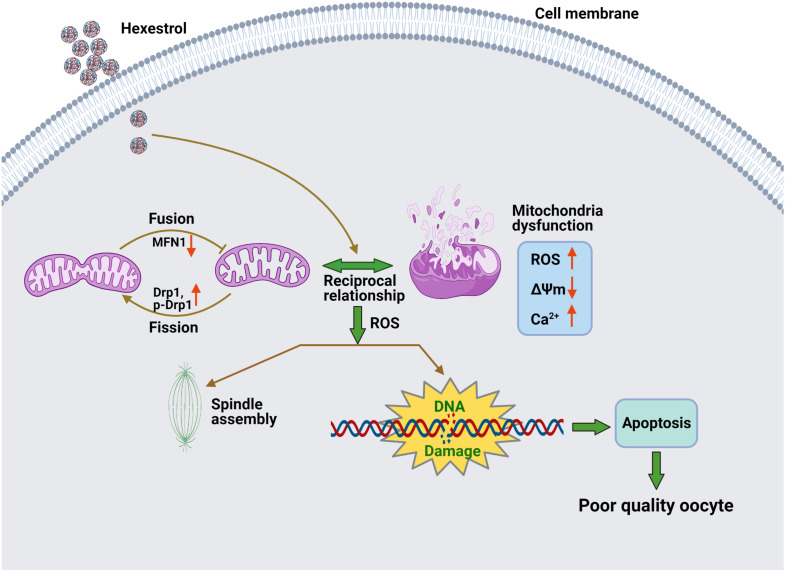
Potential mechanism of abnormal meiotic progress induced by HES exposure.

The present study demonstrated that HES exposure perturbs the balance of mitochondrial fission and fusion and results in dysfunction of mitochondria, thereby inducing oxidative stress and apoptosis in oocytes, contributing to aberrant meiotic progression (Figure 7).

## Data Availability Statement

The raw data supporting the conclusions of this article will be made available by the authors, without undue reservation.

## Ethics Statement

The animal study was reviewed and approved by the Zhejiang A&F University.

## Author Contributions

XD designed the experiments and supervised the study. DN and K-LC performed all experiments, analyzed the data, and prepared the manuscript. YW, X-QL, LL, and XM contributed to image analysis. All authors contributed to the article and approved the submitted version.

## Conflict of Interest

The authors declare that the research was conducted in the absence of any commercial or financial relationships that could be construed as a potential conflict of interest.
